# Anatomical and Histological Factors Affecting Intranasal Drug and Vaccine Delivery

**DOI:** 10.2174/156720112803529828

**Published:** 2012-11

**Authors:** Sveinbjörn Gizurarson

**Affiliations:** Faculty of Pharmaceutical Sciences, University of Iceland, Hofsvallagata 53, 107 Reykjavik, Iceland

**Keywords:** Drug delivery, intranasal administration, nasal administration, nasal anatomy, nasal cavity, nasal device, nasal histology, vaccine delivery.

## Abstract

The aim of this review is to provide an understanding of the anatomical and histological structure of the nasal cavity, which is important for nasal drug and vaccine delivery as well as the development of new devices. The surface area of the nasal cavity is about 160 cm^2^, or 96 m^2^ if the microvilli are included. The olfactory region, however, is only about 5 cm^2^ (0.3 m^2^ including the microvilli). There are 6 arterial branches that serve the nasal cavity, making this region a very attractive route for drug administration. The blood flow into the nasal region is slightly more than reabsorbed back into the nasal veins, but the excess will drain into the lymph vessels, making this region a very attractive route for vaccine delivery. Many of the side effects seen following intranasal administration are caused by some of the 6 nerves that serve the nasal cavity. The 5^th^ cranial nerve (trigeminus nerve) is responsible for sensing pain and irritation following nasal administration but the 7^th^ cranial nerve (facial nerve) will respond to such irritation by stimulating glands and cause facial expressions in the subject. The first cranial nerve (olfactory nerve), however, is the target when direct absorption into the brain is the goal, since this is the only site in our body where the central nervous system is directly expressed on the mucosal surface. The nasal mucosa contains 7 cell types and 4 types of glands. Four types of cells and 2 types of glands are located in the respiratory region but 6 cell types and 2 types of glands are found in the olfactory region.

## INTRODUCTION

The interest in intranasally delivered drugs has gradually increased over the past decades, followed by an increased interest in intranasally delivered vaccines. In recent years there has been an increasing interest in the opportunity that drugs administered to the olfactory region may be transported directly into the brain, bypassing the blood-brain barrier. Today numerous clinical trials have been scheduled, are recruiting or ongoing focusing on various aspects of nasally administered drugs and vaccines. According to the ClinicalTrials.gov registry which collect information on federally and privately supported clinical trials conducted around the world, about 18% of the trials are aimed for local treatment of the nasal cavity or the nasal mucosa. The remaining 82% focus on systemic delivery of vaccines, hormones, peptides, proteins and small molecules as well as new devices. 

In order to make any rational assessment about the usefulness of the nasal route it is important to understand the anatomy and histology of the nasal cavity. Sufficient blood flow is necessary for successful absorption of the drug and stimulation of the adrenergic nerves will decrease this blood flow resulting in significant reduction of absorption. Similarly, when irritating formulations are administered intranasally the 7^th^ cranial nerve will respond by stimulating nasal glands to secrete mucus, dilute the irritant, which will be removed rapidly to the nasopharynx and swallowed, also reducing nasal absorption.

The focus of this review is on the anatomical and histological parameters that are important for understanding the factors affecting intranasal delivery of drugs and vaccines as well as the development of nasal devices.

## EXTERNAL NOSE 

The external nose has a pyramidal shape, which may differ greatly depending on race. The variation in the shape of the nose has been widely studied by plastic and reconstruction surgeons. Although this outer structure may not have an obvious relevance for intranasal administration of drugs and vaccines, it is important for the design of devices and for understanding the administration techniques. 

Although there are several types of external noses in world population, there are mainly 3 types of nostrils; leptorrhine, described as long and narrow nostrils; plalyrrhine which are broad and flat; and mesorrhine which are between. The nose, with its central position in the face, outlined by the sharp contours of the forehead, cheeks, and jaws, is widely believed to influence decisively the observer's visual impression of the face [1]. The irregularities of nose size and shape often influence our opinion how we see the person, compared to our subjective judgment of how an ideal nose should look like.

**Fig. (1):**
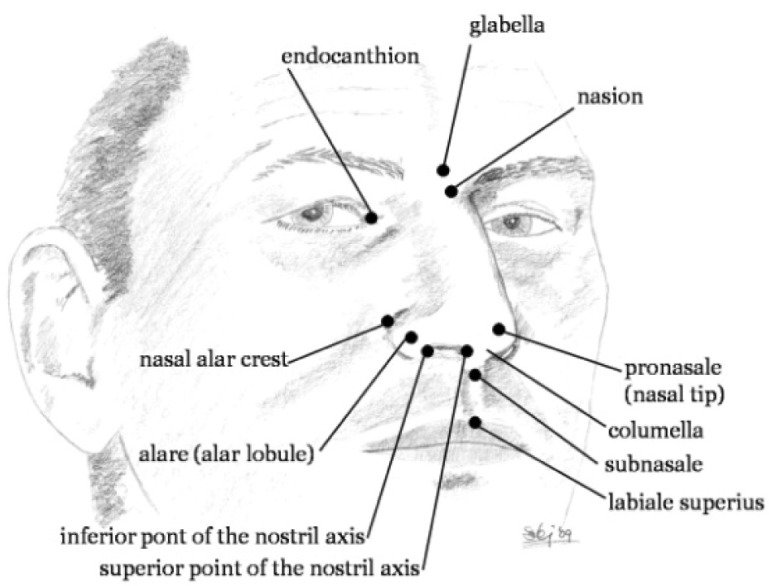
The key landmarks representing the human nose.

**Fig. (2):**
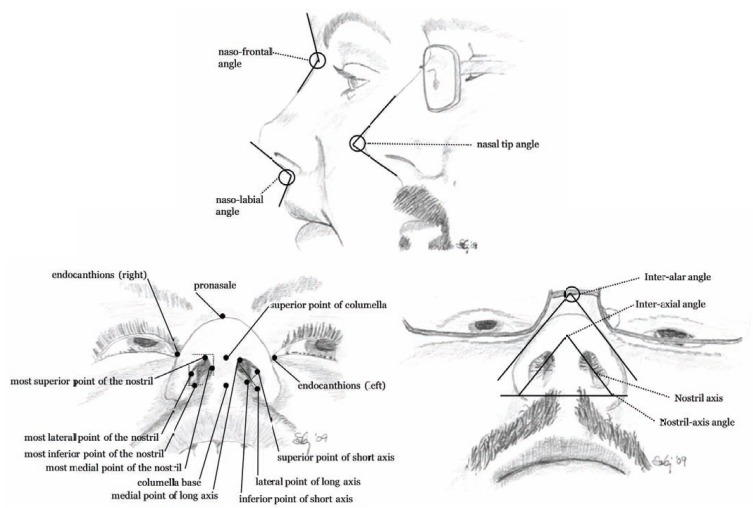
The linear and angular measurements used to analyze and characterize the external nose.

The external nasal anatomy can be separated into bony, cartilaginous, and soft tissue components. The soft tissue component of the nose is composed of skin, fibroadipose tissue and muscles of facial expression, controlled by the facial nerve. The skin is thickest over the nasofrontal angle. Reconstructive and plastic surgeons have analyzed the nose and face using linear and angular measurements of the nose and its surroundings in order to determine the major three-dimensional facial landmarks. These may be obtained into x, y and z coordinates of the nose [2, 3]. Among about 50 facial landmarks, 12 are related to the nose as shown in (Fig. **1**). These landmarks may be used to estimate facial volumes and areas by the mean of several tetrahedral and triangles [4-6]. Using the linear and angular measurements, the differences in the nose and nostrils [7] may be analyzed and characterized as shown in (Fig. **2**). 

**Fig. (3):**
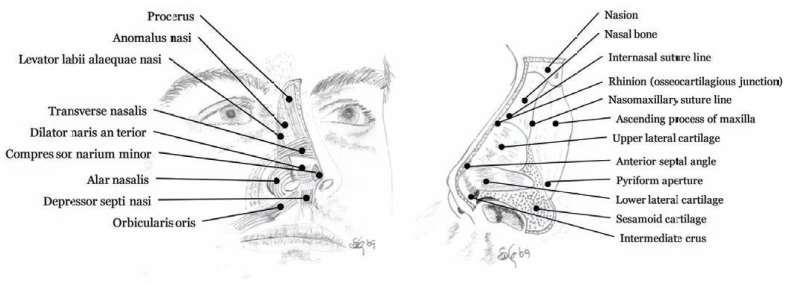
The external nasal anatomy, bones, cartilage and musculature. Procerus, levator labii alaequae nasi, and anomalous nasi represents the *elevator muscles*; alar nasalis and depressor septi nasi are the *depressor muscles*; the *compressor muscles* are constructed of transverse nasalis and compressor narium minor; and dilator naris anterior is the *dilator muscle*.

The soft tissue component of the outer nose (*nasus externus*) is composed of skin, fibroadipose tissue, and muscles (Fig. **3**). The muscles are controlled by branches of the facial nerve and dynamic motions of the facial expression and may cause some passive motions in the nose. Six of the 9 nasal muscles are located around the nostrils [8, 9]. Two muscles are especially important; dilatores nasi posterior and anterior, for they are able to widen up the entry into the nose, e.g. during heavy breathing or when nasal spray tips need to be inserted into the nasal cavity. The skin covering the outer nose contains a large number of sebaceous glands (follicles) with very distant orifices. The bony and cartilage part of the external nose builds up the pyramidal structure of the nose (Fig. **3**) including nasal septum which extends into the nasal cavity and is a crucial component of the nasal anatomy [10]. The upper lateral cartilage is attached to the nasal bone in a special way, anatomically, because cartilage does not usually articulate directly with a bone. In this case it is clinically important because it allows movement or flexion [11].

## ANATOMY

**Fig. (4):**
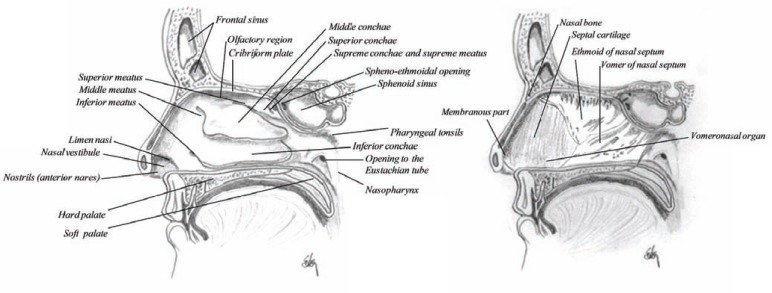
Sagittal section of the nasal cavity showing the lateral wall (or the outer wall) of the nasal cavity on the left and the inner wall (nasal septum) on the right. The septum shows deep groves for the vascular nervous canals.

The nose is divided into two large irregular cavities formed by 14 bones connected to each other by a tough fibrous membrane, which structure a roof, a floor, an inner and an outer wall. Each cavity extends from the base of the cranium to the roof of the mouth and opens to the face through the nostril (anterior nares) and extends to oval opening into the upper part of the pharynx (throat), called nasopharynx (Fig. **4**). The nasal passage is about 12-14 cm long and about 5 cm high. The total surface area of both nasal cavities is about 160 cm^2^ (96.000 cm^2^ if the nasal epithelial microvilli are included), the total volume is about 15 mL [12].

**Fig. (5):**
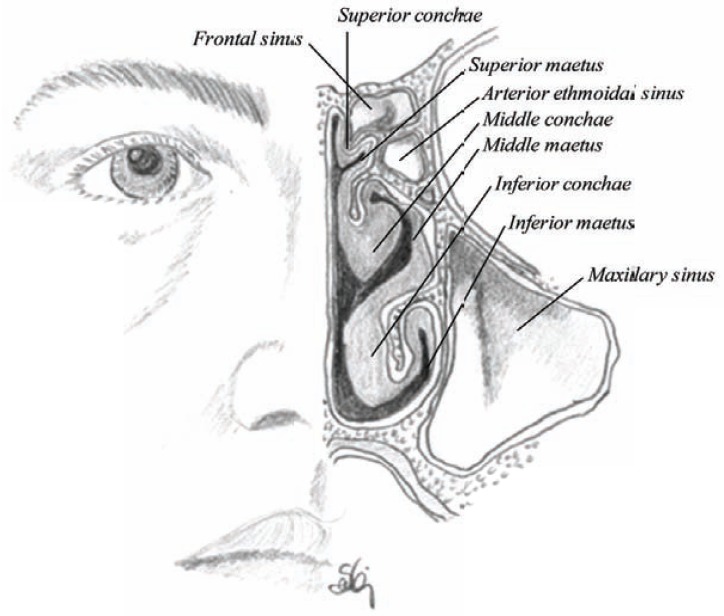
Horizontal section through the main nasal passage showing the nasal septum, folding of the conchae and the nasal passage in relation to the paranasal sinuses.

**Fig. (6):**
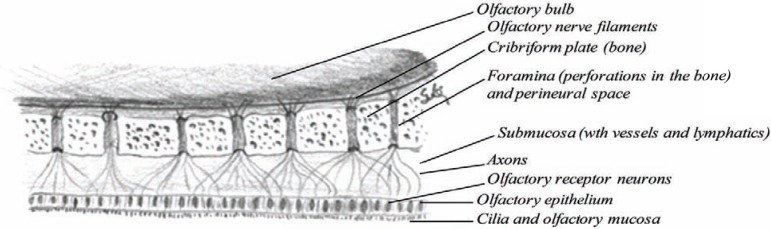
The anatomy of the cribiform plate (olfactory region).

The nasal cavity is much narrower above than below, where the olfactory region is located (Fig. **5**), which is about 2.5 cm^2^ in each cavity (about 3000 cm^2^ in both cavities if the microvilli are included in the calculations) or about 3% of the nasal surface area. Table **1** summarizes the anatomical facts of the nose. The horizontal bone separating the nasal cavity and the brain is called the cribriform plate of the ethmoid [8], a highly perforated bone by small vascular apertures that provides access for the nerve endings to enter the outer surface (Fig. **6**). These perforations are called foramina and are 20 on each side of the nose. This is the only site in the body where the central nervous system is in direct contact with the outer surface (mucosal membrane). The nasal floor is much wider than the upper (olfactory) region, concave in structure and wider in the middle than at either opening (Fig. **5**). 

The nasal septum, also called the inner wall, separates the two nasal cavities. This wall is thickest in the superior border and has deep grooves, marked by numerous vascular and nervous canals providing pathway for e.g. the nasopalatine nerve (Fig. **4**). The septum is much thinner in the middle than at the circumference and is generally bent to one or the other side. The posterior border of the nasal septum is free and separates the nasal cavities from behind [8]. It has been shown that the septum cartilage increases rapidly in size during the first years of life and remain constant after the age of two years [13]. However, ossification of the cartilaginous septum begins after the first six months of life and continues until the age of 36 [14]. This change may explain frequently encountered septal deviations in adults, if it cannot be explained due to trauma. These deviations have also been shown to prevent successful delivery of drugs into the nasal cavity [15]. On either side of the anterior nasal septum, there is a small bar of cartilage, the vomeronasal cartilage. This is associated with a small opening which leads into the rudimentary vomeronasal organ (of Jacobson). It is usually a simple short tubular sac, from 2-6 mm in length, lined by epithelium similar to the surroundings. 

The outer wall, however, is further convoluted into the folds of conchae or turbinate, which engender increased resistance to the airflow, producing intimate contact between inspired air and the mucosa. There are three (or four) conchaes in each cavity: the superior, middle and inferior conchae producing three irregular passages inside the nasal cavity called superior, middle and inferior meatuses (Figs. **4** and **5**) [9, 16-19]. 

The superior conchae and meatus is the smallest of the three and it is situated at the upper and back part of the nasal cavity. Rarely mentioned in anatomical textbooks is the fourth conchae, which are above the others and much smaller than the superior conchae, known as supreme conchae. It is only found in about 60% of the population [11]. The opening into the sphenoidal sinuses is located above and behind the superior conchae [8].

**Fig. (7):**
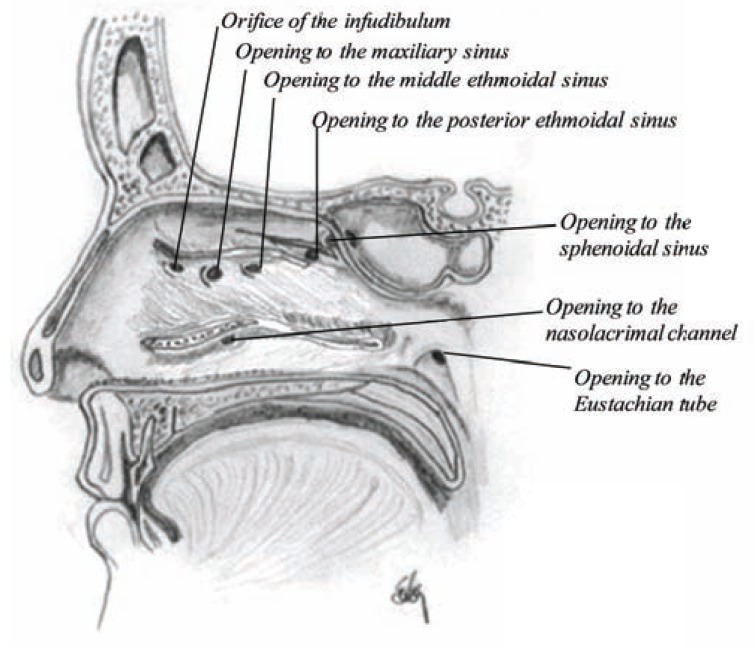
Sagittal section showing the openings into the nasal cavity. The inferior, middle and superior conchae have been cut over to show the location of each opening.

The middle concha is much larger than the superior one and is located between the superior and the inferior conchae. In front of the middle concha there is an opening called orifice of the infudibulum that goes through a long flexuous canal, the infudibulum, into the frontal sinuses, which is connected to the anterior ethmoidal sinuses from above and middle ethmoidal sinuses from below. The middle ethmoidal sinuses also open into the middle meatus. The posterior ethmoidal sinus, which occasionally communicates with the sphenoidal sinuses, opens into the superior meatus. The maxillary sinuses are two large pyramidal cavities which enter into the nasal cavity through a large opening anteriorly into each of the middle meatuses (Fig. **7**) [8, 9].

The inferior conchae and meatus are the largest of the three and extend along the entire length of the outer nasal wall. Anteriorly, there is an opening called orifice of the canal for the nasal duct, a long channel into the nasal duct and further through the lachrymal groove into the lachrymal duct. The channel is narrower in the middle, but wide in both ends, especially where it exits into the nasal cavity [8]. The supreme meatus, however, is usually a barely perceptible furrow below the supreme conchae, when present.

The nasal vestibule has the smallest cross-sectional area in the respiratory tract (approximately 0.3 cm^2^ on each side) and extends from the entrance of the nostrils, lined with skin containing vibrissae or hairs and sweat and sebaceous glands. The vibrissae guard the anterior ends of the inferior conchae and there is a gradual transition from the skin of the vestibule to the mucous membrane of the remainder of the cavity. 

As shown in (Fig. **7**) and described here above, there are seven openings into the nasal cavity (excluding the nostrils and the throat) where most of them drain into the middle meatus except tears which drain into the inferior meatus and the fluid from the ear that drains into the nasopharynx, through an opening into the eustachian tube, a wide and about 35 mm long channel into the inner ear. The group of openings into the middle meatus is also called hiatus semilunaris, which means a half moon-shaped opening due to the many openings into that space.

The conchae and the meatus are responsible for expanding the surface area of the nasal cavity, providing an area where most significant drug absorption takes place [12].

## SINUSES

The paranasal sinuses are pairs of air filled cavities covered with a thin layer of respiratory mucosa and named after the skull bones: frontal, ethmoid, maxillary and sphenoid. The frontal sinuses grow in size until the late teens. They open via the orifice of the infundibulum into the anterior part of the middle meatus (Fig. **7**). The ethmoid sinuses (anterior and posterior) have reached their size at the age of 12 years. They drain into the middle and superior meatus. The maxillary sinuses are well established at birth and grow until 12 years of age. At that time they may have a volume of around 15 mL. Like the frontal sinuses, the maxillary sinuses drain into the middle meatus. Around 25-30% drains through the infundibulum, but others through the opening to the maxillary sinuses. These sinuses were first described by Leonardo da Vinci [20]. The sphenoid sinuses are very small at birth until about 7 years. But at late teens they have reached their size, although they may continue growing in adults. They drain into an opening above the superior chonchae. 

The functions of the sinuses are not really understood. One is that they form a collapsible framework to protect the brain from trauma, where other theories describe their ability to provide thermal insulation for the brain, imparting in voice resonance, humidifying and warming inspired air and provide moist to the nose.

## ARTERIES

The nasal cavity is supplied with arteries, veins, lymphs and neurons as shown in Table **2**. The vascular supply to the outer nasal region is provided by the superior coronary and lateral nasal artery. Superior coronary supplies the upper lip and provides two vessels into the nose: inferior artery of the septum, which supplies blood to the anterior part or the nasal septum; and artery of the ala, which supplies the ala of the nose. The rest of the nasal muscles and outer nose is supplied by lateral nasal artery. 

**Fig. (8):**
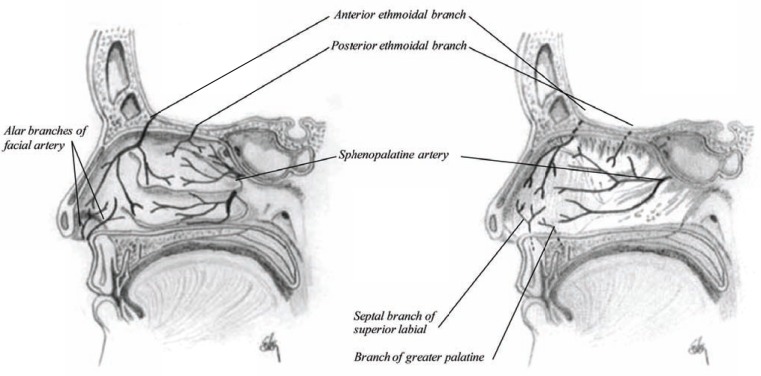
Sagittal section of the nasal cavity showing the arterial supply to the lateral wall and the nasal septum.

The nasal cavity, however, receives its blood supply from three sources (Fig. **8**). The predominant supply is from one branch of the sphenopalatine artery, which enters the cavity behind the superior meatus and divides into two branches: one internal, called artery of the septum and passes along the nasal septum and supplies the mucous membrane with fresh blood. The others are two external branches supplying the mucous membrane of the outer wall, conchaes, as well as the ethmoidal and sphenoid sinuses. The nasal cavity also receives arterial blood from ethmoidal branches of the ophthalmic artery that enter the nasal cavity through openings in the cribriform plate and this artery supplies fresh blood to the anterior part of the inner and outer wall of the nose as well as the ethmoidal sinuses and the frontal sinuses. The anterior ethmoidal artery is accompanied by the nasal nerve into the nasal cavity [21]. The direction of arterial blood flow is anteriorly against the flow of air, which helps in warming incoming air. The blood supply is particularly rich on the septum where the squamous epithelium becomes respiratory epithelium or the Schneiderian membrane, a region called Kiesselbach’s plexus (a capillary loop of 1.5 mm^2^), where the majority of nosebleeds come from [9]. 

In contrary to the ethmidal and sphenoid sinuses, the frontal sinuses receive blood from the supraorbital and supratrochlear arteries which are branches of the ophthalmic artery and the maxillary sinuses receive blood supply from the maxillary artery and the facial artery.

## VEINS

**Fig. (9):**
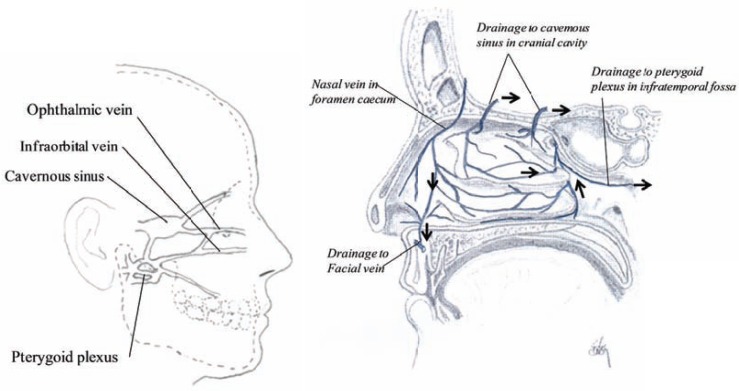
Sagittal section showing the major connections of the venous draining from the nasal cavity (left) and sagittal section of the nasal cavity showing the venous drainage in the lateral wall (right).

The veins of the nose are valveless and begin in a venous plexus on the inferior nasal conchae, inferior meatus and the back part of the septum and drain into the pterygoid plexus. The ethmoidal veins join the ophthalmic plexus and proceed to the cavernous sinus (cavernous plexi) as shown in (Fig. **9**) [11, 14, 21, 22]. Their network and structure of the veins correspond approximately to the arterial structure (Fig. **8**). The veins from the upper nasal cavity drain into veins in the interior of the skull, through the foramina in the cribriform plate, the foramen cœcum and collected into the superior longitudinal sinus. Here, the sinuses are venous channels, only found in the interior of the skull [21, 23, 24]. The cavernous sinus is able to change its blood volume in response to neural, mechanical, thermal, psychological or chemical stimuli. The endothelium and the longitudinal muscles may therefore influence the blood stream through the nasal cavity.

**Fig. (10):**
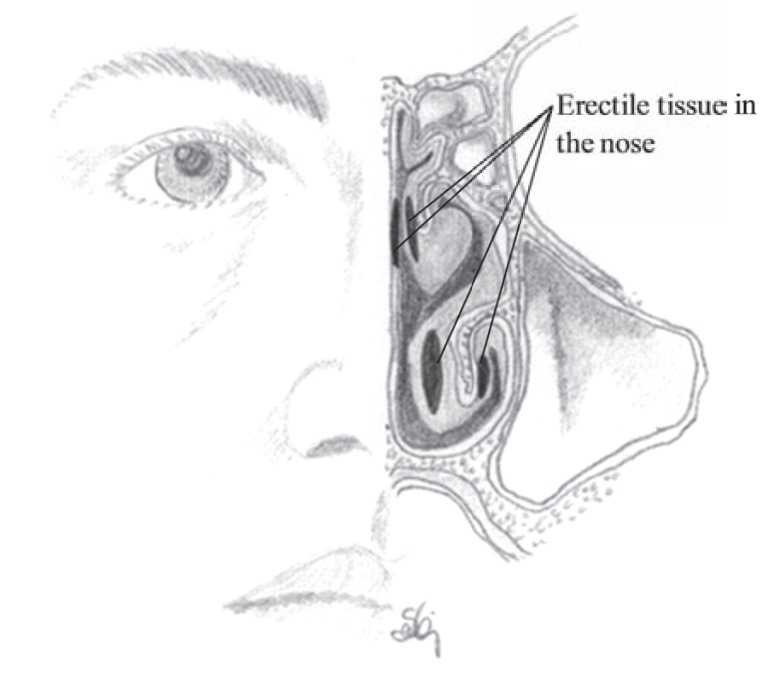
The location of the erectile tissue inside the nasal cavity.

The nasal mucosa is highly vascular with a network of cavernous or fenestrated veins beneath the mucous membrane. The fenestrae always face the respiratory epithelium and are believed to be one of the sources of fluid for humidification. The epithelium is also supplied with a dense network of erectile tissue, which is also cavernous and particularly well developed over the conchae and septum as shown in (Fig. **10**). The vascular bed is especially high in density over the lower part of the septum and over the middle and inferior conchae, which provides a promising condition for drug absorption. Due to the large veins the lamina propria and the mucosa is particularly thick over the surfaces of the middle and inferior conchae. Constriction of the blood vessels that decrease blood flow and blood content in the nasal mucosa may therefore affect the rate of absorption, whereas vasodilatation may result in the opposite response [12]. The penetration of drug through the mucosa is therefore highly influenced by the blood flow in the region under normal and pathological conditions.

**Fig. (11):**
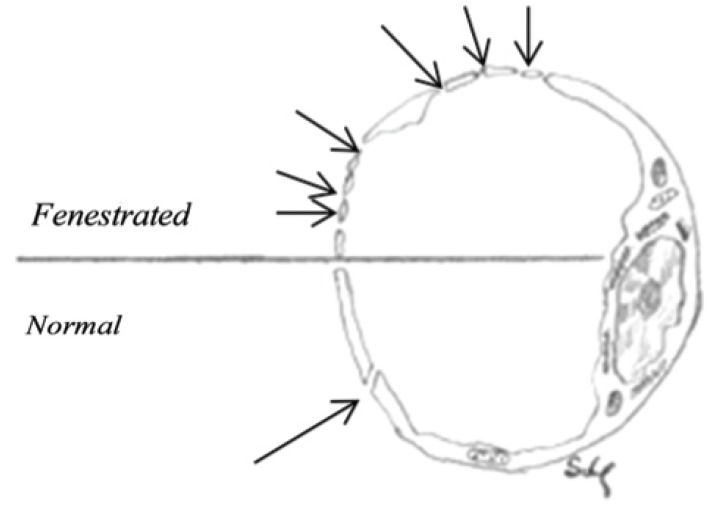
Cross section of a normal vein and a fenestrated vein. The drawing demonstrating the absorption capacity of nasal venous system.

The structure of the blood vessels of the nasal mucosa is of major importance for the function of the nose. The capillaries are fenestrated and the porosity of the endothelial basement membrane is one of the characteristics of the nasal blood vessels as shown in (Fig. **11**).

## LYMPHATICS

**Fig. (12):**
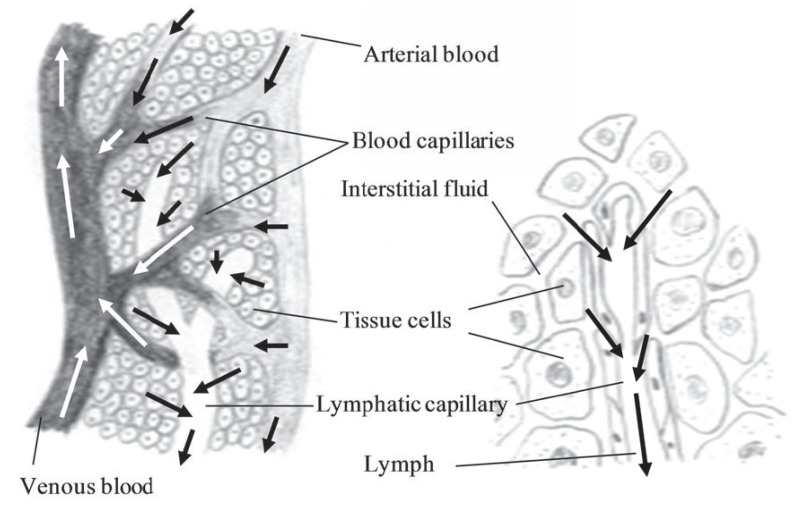
The relationship between the lymphatic system and the blood capillaries as well as the drainage of fluid into the lymphatic system.

The lymphatic system includes lymphatic vessels and glands through which they pass. The lymphatics have the property of absorbing materials from the tissues and conveying them into the circulation as shown in (Fig. **12**). Lipids and lipid soluble compounds are examples of compounds absorbed by the lymphatic system. Due to the watery fluid (called lympha), the lymphatics are delicate transparent vessels. The lymphatic vessels begin as close-ended vessels called lymphatic capillaries and are found throughout the nasal mucosa (Fig. **12**). 

As blood capillaries, lymphatic capillaries merge to form larger tubes, lymphatic vessels, but at intervals along the lymphatic vessels, the lymph flows through lymphatic tissue structures called lymph nodes. The lymph nodes are oval or bean-shaped structures covered by a capsule and divided into compartments or sinuses and many follicles packed with lymphocytes. The lymph flows through the nodes in one direction. It enters through the afferent lymphatic vessels and exit through the efferent lymphatic vessels. 

**Fig. (13):**
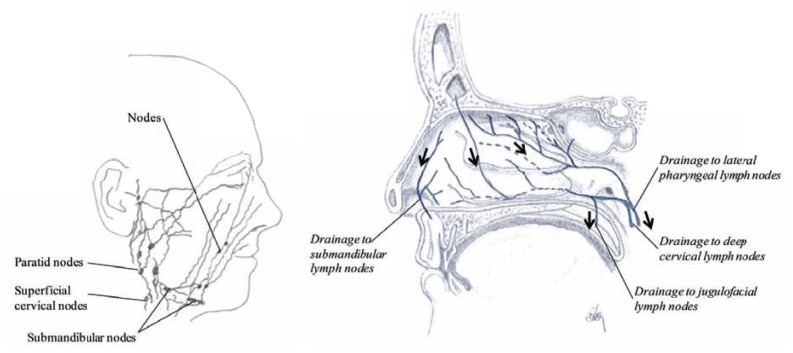
Drawing showing the lymphatic draining from the nasal region (left) and a sagittal section of the nasal cavity showing the lymph drainage in the lateral wall (right).

Lymphatics from the anterior part of the nose drain through the nostrils with lymphatics of the skin to submandibular lymph nodes, but in the middle and the posterior part of the nasal cavity the lymph drainage is mainly to the lateral pharyngeal lymph nodes, the deep cervical lymph nodes and to the jugulofacial lymph nodes as shown in (Fig. **13**). The buccal nodes and the mandibular nodes collect drainage from the mucous membrane of the nose and bring it further to the submandibular nodes (Fig. **13**). The nose-associated lymphoid system (NALT) around the nasal and buccal cavity, which are a combination of occasional M-cell clusters or lymph corpuscles and the adenoid tissue (as well as the tonsils in the pharynx) has been called Waldeyer’s ring. Due to this lymphoid tissue, polyps may be found growing from the outer wall of the nasal cavity.

In the olfactory region there is an anatomical relationship between the olfactory nerves and the extracranial lymphatics through the foramina in the cribriform plate. The lymphatics form a collar around the root of the olfactory nerve within the cribriform plate, so there is very limited leakage or drainage of cerebrospinal fluid into the nasal submucosa (Fig. **13**). In addition, the lymphatics are able to guard the entrance from the nose to the brain, taking part in the defense system by protecting the brain from slowly moving molecules from being transported from the nasal cavity. 

## NERVES 

**Fig. (14):**
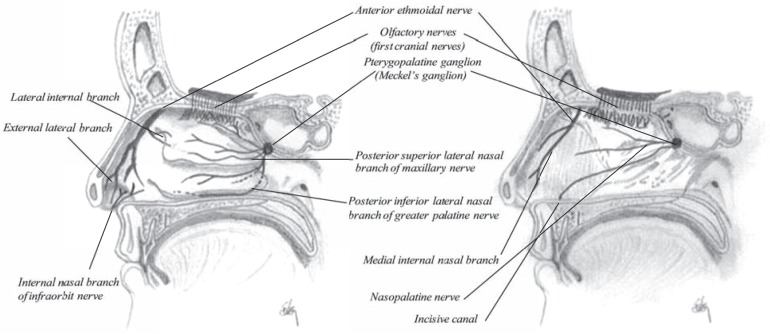
Sagittal section of the nasal cavity showing the neuronal innervations, to the lateral wall and the nasal septum.

The largest cranial nerve, so-called fifth nerve or trifacial nerve (nervous trigeminus) of the head and face supports the nasal cavity. This nerve is mainly a sensory nerve in addition to a number of other functions. The first division of this fifth nerve is called the ophthalmic nerve (nervous ophthalmicus), it supplies the eyeball, the lachrymal gland, the frontal sinuses, the nasal cavity and the integument of the nose [25]. The ophthalmic nerve divides into three branches just after passing through the sphenoidal fissure, where one is the nasal nerve, a deeply placed nerve that enters the cavity through the anterior ethmoidal foramen and via a shallow opening on the front of the cribriform plate (Fig. **14**). After entering into the nasal cavity it divides into two branches, an internal and an external branch. The internal branch supplies the mucous membrane near the anterior part of the septum of the nose. The external branch supplies the mucous membrane covering the anterior part of the outer wall of the nostrils. When it leaves the cavity it supplies the tip of the nose and joins the facial nerve. 

The ganglion supporting nerves to the nasal cavity is the pterygopalatine ganglion or the Meckel’s ganglion (Fig. **14**). Four branches enter from Meckel’s ganglion where two of them supply the nose: the internal branch to the nose and the posterior branch to the nasal cavity and the pharynx. The ascending branch supplies the mucous membrane of the sinuses such as posterior ethmoidal sinuses and the sphenoidal sinuses. The descending or palatine branch supplies the lining membrane of the nose. 

The nasopalatine nerve, a branch of the maxillary nerve, courses with the sphenopalatine artery to the septum, the posterior parts of the conchae (as posterior lateral nasal branches) and the palate (as greater and lesser palatine nerves). The anterior ethmoidal nerve, a branch of the ophthalmic nerve, courses with the anterior ethmoidal artery to the anterior parts of the conchae. Branches of the intraorbital nerve, a branch of the maxillary nerve, enter the nasal cavity from the face. The maxillary sinuses are innervated by branches of the maxillary nerve as well as parasymphathetic fibers from the facial nerve.

All the muscles in the nasal region are controlled by the facial nerve or the seventh cranial nerve. This nerve is responsible for muscles of facial expression but also it affects the nasal glands via parasympathetics. The supporting cells of the olfactory glands also called Bowman’s glands are innervated by branches of the facial nerve. Injury or even odor may irritate the facial nerve in the olfactory epithelium. If the injury is severe it may cause paralysis of the facial muscles called Bell’s palsy [26]. Impulses generated in the nerve, stimulate the lachrymal glands in the eyes and the nasal mucous glands. Examples are irritation to the mucosal membrane, which leads to vasodilation and increased secretion by its glands, and may be followed by sneezing. Sneezing, however, involves voluntary muscles and may be inhibited voluntarily.

Adrenergic neurons mediate vasoconstriction but cholinergic ones cause vasodilatation and secretion. Nasal mucosal blood vessels are surrounded by adrenergic nerves, in which α-adrenoreceptors show a functional predominance. Stimulation of these receptors produces a decrease in both blood content and blood flow in the nasal mucosa. 

### The Olfactory Nerve (First Cranial Nerve)

**Fig. (15):**
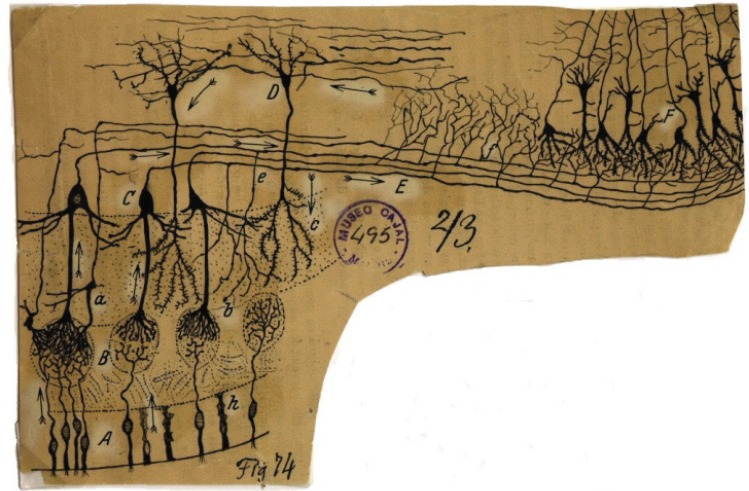
Olfactory receptor neurons in the olfactory epithelium. A reproduction of an original drawing made by Cajal in 1894. Reprinted by permission from the Cajal Institute, Spain.

The nasal cavities receive both sensory and visceral innervations. Small olfactory nerves enter each nasal cavity through openings in the cribiform plate of the ethmoid. These nerves are bundles of axons derived from small cell bodies in the epithelia on either side of the septum and on lateral walls near the roof of the nasal cavities (Fig. **6**). The nerves serving the olfactory region are called the first cranial nerves or the olfactory nerves. These are twenty special nerves [27]. They are connected to the olfactory bulb, an oval grayish mass which rests on the cribiform plate of the ethmoid bone. Each nerve divides into two sites after entering the nasal cavity: the larger part is spread out over the upper third of the septum and the smaller part on the outer wall and is distributed over the superior conchae and on the surface in front of the superior conchae. The olfactory nerves are made by non-medullated fibers and are not protected by the white substance of Schwann. They convey nerve impulses arising from the bipolar olfactory neurons in the olfactory mucosa and through the axons the impulse is brought to a synapse in the olfactory bulb, as presented by Dr. Cajal in 1894 in (Fig. **15**). 

### Vomeronasal Organ

**Fig. (16):**
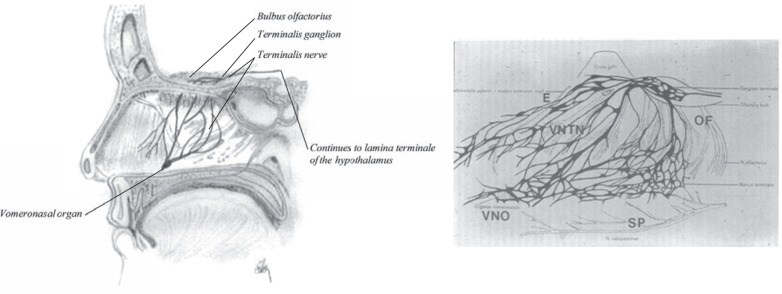
Sagittal section of the nasal cavity showing the vomeronasal organ (left). To the right is a drawing of the nasal septum containing the vomeronasal organ, by Dr. C. Brookover in 1914.

The vomeronasal organ is another chemoreceptor organ found in the nasal cavity. It detects external chemical signals and is connected to the terminalis nerve to the brain, through the vomeronasal-termianlis nerve (Fig. **16**) [28]. The vomeronasal organ is essential for promoting bonding between newborn and mother [29], eliciting and maintaining male and female sexual behaviour, influencing the onset of puberty, the oestrus cycle, gestation, maternal behaviour and social behaviour [30-32]. It also influences mood changes in woman [33]. In adults the vomeronasal organ is a tube-like membranous organ, 2-10 mm long, located as shown in (Fig. **16**). It opens into the nasal cavity through a small orifice (about 1 mm in diameter) as a small depression of the nasal mucosa, close to the base of the nasal septum.

The nerves are unmyelinated with a small diameter that branch from the vomeronasal-terminalis nerve, surrounding the vomeronasal area and enters the cranium through the cribiform plate and runs parallel with the olfactory bulb and the olfactory tract to end in the lamina terminalis of the hypothalamus. This connection provides important connections between the nasal cavity (vomeronasal organ), the hypothalamus and the limbic system [34]. Apically to the vomeronasal organ is a mucus gland producing proteins and mucus that drain in the vomeronasal organ [35].

## GLANDS

Anteriorly in the nose, are about 200-300 glands that produce watery secretion containing proteins and electrolytes, which may be seen using a magnifying-glass. These are small scattered serous glands, the structure of which resembles that of the serous salivary glands (parotid gland) with a large opening (0.1-0.4 mm in diameter). This fluid is commonly visible in cold weather as droplets hanging from ones nose. 

**Fig. (17):**
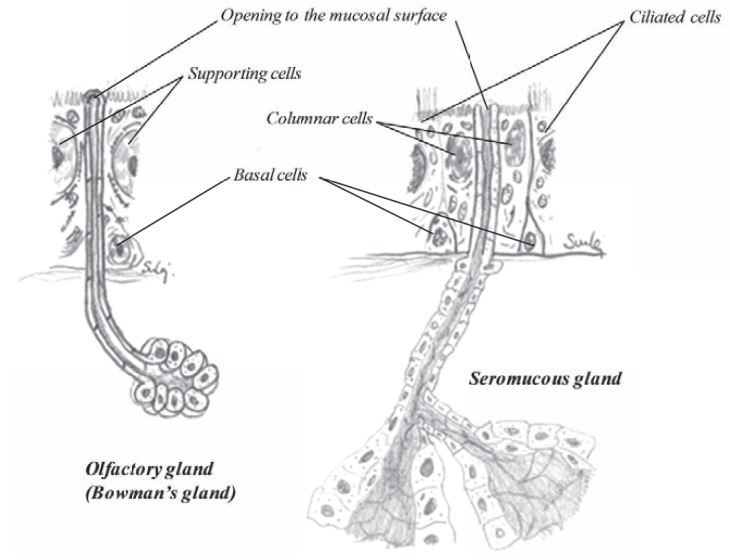
The anatomy of a olfactory gland (left) and a seromucous gland (right).

In the other part of the nasal cavity there are numerous seromucous glands (Fig. **17**), secreting proteineous secretion where large amounts of mucus are added. The density of glands differs depending on age and location. At birth there are about 34 glands per mm^2^. When growing, the area of the nasal cavity increases and the density decreases, being around 17-18 glands per mm^2^ at age one, 13 glands/mm^2^ at age 5 and about 8-9 glands per mm^2^ for adults, which equals to about 10,000 glands on the nasal septum, alone [36] and they are spread in similar matter as the goblet cells. Around 8.5 glands/mm^2^ in the anterior part to around 7.2 glands/mm^2^ in the posterior part [36]. Similarly, the density on the conchae falls from 8.4 glands/mm^2^ on the anterior middle conchae down to 7.2 glands/mm^2^ in the posterior part. The sinuses contain very low density of glands, from 0.06 gland/mm^2^ in the sphenoidal sinus and 0.08 gland/mm^2^ in the frontal sinus to 0.20 gland/mm^2^ in the maxillary sinus and 0.47 gland/mm^2^ in the ethmoidal sinus. The total number of glands in the nasal cavity has been calculated to be around 90.000 [36]. The secretory control of the glands is under the parasympathetic nervous system [37]. 

In the olfactory region there are numerous olfactory glands, called Bowman’s glands (Fig. **17**). These are tubuloalveolar serous glands that deliver watery, although proteineous secretions onto the olfactory surface. Their secretion is important for the function of the olfactory region, both as a trap but also as a solvent for odoriferous substances. Constant flow from the glands keeps the mucosa clean so that new scents can be continuously detected as they arise.

The vomeronasal organ is also supplied with tubuloalveolar serous glands that empty their secretions into the vomeronasal organ lumen via short ducts around the vomeronasal epithelium [38]. These glands produce substances required for facilitating reception of chemical stimuli and chemoreception as well as secretions for dissolving chemicals [39]. The glands associated with vomeronasal organ are not fully understood [40].

## HISTOLOGY

**Fig. (18):**
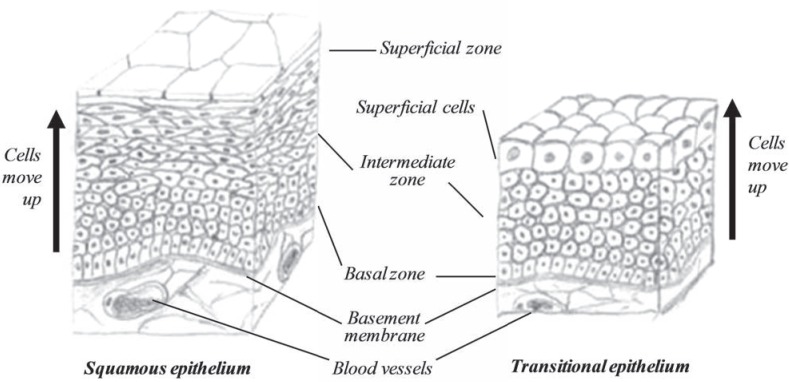
The structure of a squamous (left) and transitional (right) epithelium.

About ⅔ of the anterior vestibule and nostrils are covered by skin, containing large stiff hair called vibrissae that guard the entrance into the nasal cavity by collecting airborne dust and bacteria. About ⅓ is covered by a squamous epithelium anteriorly, followed by transitional epithelium (Fig. **18**).

In the remainder of the nasal cavity there are mainly two types of epitheliums [12, 18]. The main epithelium is a typical respiratory epithelium also called Schneiderian membrane, ciliated pseudostratified columnar epithelium with goblet cells scattered throughout, lining most of the respiratory tract and the sinuses. The second is the olfactory epithelium, a chemoreceptive type of epithelium which is thinner and only found in the upper part of the nose and the vomeronasal organ. Another type of tissue may also be found in the nasal cavity, which is the lympoid tissue, where occasional M-cells or M-cell clusters (lymph corpuscles) may be found in the epithelium. Their main density is posterior in the adenoid tissue. The epithelium covering the paranasal sinuses is similar to respiratory mucosa, but less vascular, thinner, and more loosely attached to the bony walls [11]. 

The nasal epithelium rests on a well defined layer between the epithelium and the connective tissue, made of tissue fibrils or collagen fibrils, called basement membrane. The epithelial cells are anchored to this extracellular matrix. The underlying connective tissue is called lamina propria, a tissue containing dense network of fenestrated capillaries, supplying the tissue with large volume of blood, which drain directly into the systemic circulation, avoiding first pass metabolism for compounds entering the body through the nasal route [41]. The lamina propria also prevents the epithelium from dehydration due to numerous small glands (and the goblet cells embedded in the epithelial sheet) producing serous and mucous secretions for the epithelium [42]. Their production keeps the epithelial surface moist and provides a fluid sheet that traps foreign materials. The ciliated cells then move the moist sheet or the mucus, as a whole, the beating of the cilia being unidirectional. The basement membrane is thickened in patients with rhinitis [43]. In the olfactory region, the lamina propria is directly contiguous with the periosteum of the underlying bone and contains numerous lymphatic vessels, unmyelinated olfactory nerves, myelinated nerves, olfactory glands (branched tubuloacinar serous glands) in addition to dense network of capillaries.

The mucosal membrane is thickest over the conchaes and the center of the septum due to highly vascularised mucosa which is also linked to the erectile tissue in the nose, particularly in the middle and inferior chonchae as shown in (Fig. **10**), which enables the airways to widen or narrow. This autonomically controlled vasculature of the nasal tissue, in combination with its rich supply of secretory cells is of importance in the modification of inspired air. 

The olfactory epithelium is found in the olfactory region of the nose and in the vomeronasal organ. This part of the nasal cavity is specialized for chemoreception, it is also ciliated, but they are far less numerous than in the respiratory region and there are no goblet cells in that region. There are five cell types present there, where one is chemoreceptive. As in the respiratory region, there are serous glands in the lamina propria under the olfactory region called olfactory glands or the Bowmanns glands. Secretions from these clean the sensory structures and ready them for reuse. They secret aqueous non-viscous fluid that has appropriate characteristics to solubilise substances for olfaction and clean the olfactory cilia between smells.

The nasal mucosal consists of seven types of cells, (Table **3**), where four of them are found in the respiratory region and other five in the olfactory region. Their density and distribution differs greatly from one region of the nose to another [16].

### Ciliated Cells

**Fig. (19):**
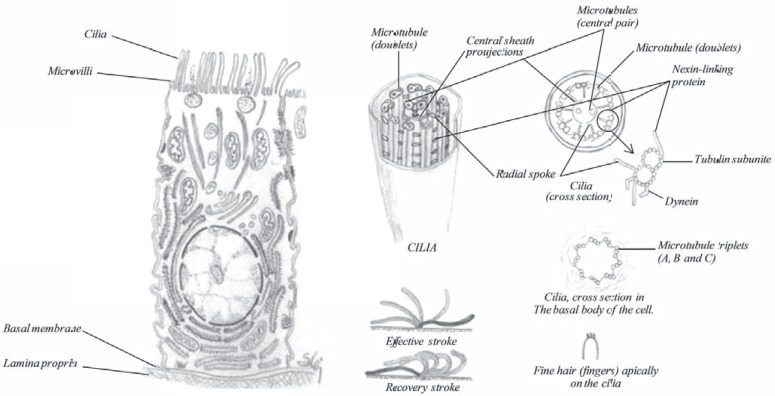
The anatomy of a ciliated cell and schematic diagrams showing the molecular structure of cilia.

The ciliated cells (Fig. **19**) are the most frequent cell type in the nasal cavity, their function is e.g. to provide a coordinated sweeping motion of the mucus coat to the throat, called ciliary escalator or ciliary clearance. This function is an important protective mechanism for removing mucus and trapped inhaled particles. Due to this clearance, inhaled drugs have a short window to be absorbed, otherwise they will be cleared to the throat and swallowed. Numerous mitochondria are found in the cytoplasm in the apical part, as signs of an active metabolism. All ciliated cells are covered with about 300 microvilli, which are fingerlike cytoplasmic expansions on the surface of the cells in addition to the cilia. These microvilli are uniformly distributed on the entire apical surface [16], increasing the surface area significantly. The microvilli also prevent drying of the surface by retaining moisture essential for ciliary function.

Each ciliated cell contains about 100-250 mobile cellular appendage called cilia, 0.3 µm wide and 5 µm in length as shown in (Fig. **19**). Their function is to help transporting fluid or the carpet of mucus towards the throat where after it is swallowed. The cilia contain a motor protein called dynein and microtubules, which are composed of linear polymers of globular proteins called tubulin. The core of each of the structures is termed the axoneme and contains two central microtubules that are surrounded by an outer ring of nine doublet microtubules. One full microtubule and one partial microtubule, the latter of which shares a tubule wall with the other microtubule, comprise each doublet microtubule. The dynein molecules are located around the circumference of the axoneme at regular intervals along its length where they bridge the gaps between adjacent microtubule doublets. Dynein, the motor protein, uses the energy of adenosine triphosphate (ATP) hydrolysis to move along the surface of the adjacent microtubule. The ciliary activity is based on the movement of the doublet microtubules in relation to one another, initiated by the dynein arms. Hydrolysis of ATP produces a sliding movement along the microtubule, where the dynein molecules produce a continuous shear force sliding toward the ciliary tip (Fig. **19**), a movement called the effective stroke. At the same time, a passive elastic connections provided by nexin and the radial spokes accumulate the energy necessary to bring the cilia back to the straight position (recovery stroke).

A plasma membrane surrounds the entire cilia, which is attached to the cell at a structure termed the basal body (also known as a kinetosome). The basal bodies maintain the basic outer ring structure of the cilia, but each of the nine sets of circumferential filaments is composed of three microtubules.

The ciliary motion is often described as whip-like, or compared to the breast stroke in swimming. Adjacent cilia move almost simultaneously (but not quite), so that in groups of cilia, wave-like patterns of motion occur.

**Fig. (20):**
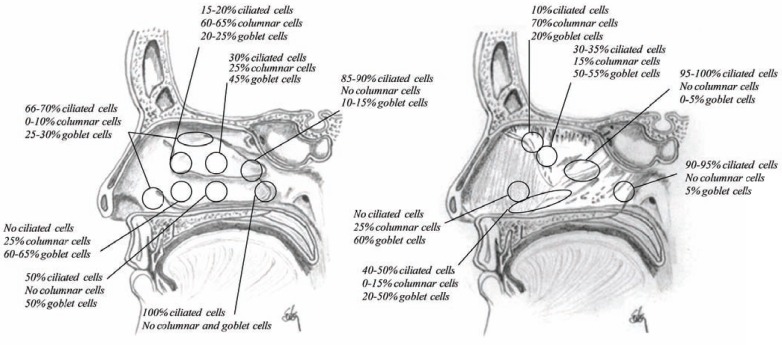
Sagittal section of the nasal cavity showing the distribution of ciliated, columnar and goblet cells.

Halama *et al.* [44] studied the density and distribution of epithelial cells in the human nasal mucosal using scanning electron microscopy. They showed that the anterior one third of the nasal cavity is non-ciliated but the cilia start occurring just behind the front edge of the inferior turbinate and the posterior part of the nasal cavity as well as the paranasal sinuses are densely covered by cilia. The distribution pattern of ciliated cells corresponds well with a map of nasal airflow indicating that the density of ciliated cells is inversely proportional to the linear velocity of inspiratory air in the nasal cavity (Fig. **20**). Consequently there are less cilia in the upper part of the nasal cavity than along the floor. Low temperature (in some cases very strong current of cold air with low humidity), low humidity and polluted air may also contribute to a reduced number of ciliated cells in the anterior part of the nasal cavity. However, newborn and laryngoectomized subjects have cilia in the entire nasal cavity [16]. The current of air contribute highly to the clearance from the nasal cavity and if that current is eliminated e.g. due to obstruction in one site of the nose, the side where normal airflow is obtained show normal distribution of ciliated cells, where the obstructed side show dense cilia.

### Basal Cells 

**Fig. (21):**
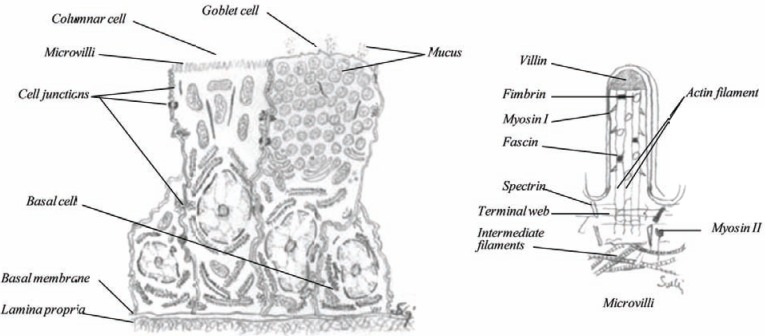
The anatomy of a basal cell, columnar cell and a goblet cell (left) and a schematic diagram of a microvilli (right).

Basal cells (Figs. **20** and **21**) serve as a reserve population by maintaining individual cell replacement in the epithelium. They carry necessary information on each of the other cell types and lie on the basement membrane. These cells do not reach the airway lumen but due to their pluripotency they are able to grow and become the required cell type. Basal cells tend to be prominent because their nuclei form a row in close proximity to the basal membrane.

### Columnar Cell, Non-Ciliated

The columnar cells (Fig. **21**) are like ciliated cells, on the basement membrane and stretch to the airway lumen where they bear microvilli. The microvilli are slender fingerlike cytoplasmic expansions of the cell membrane that have the capability to absorb compounds. It has been shown that a number of drug substances, proteins are absorbed through the columnar cells. The influenza virus also uses this cell type as a port for crossing the nasal mucosal. Due to the microvilli, the surface area of the cell may be increased over 600 times [45-47]. The columnar cells contain numerous mitochondria in the apical part, as signs of an active metabolism. 

### Goblet Cells

Goblet cells (Fig. **21**), also called mucus cells or chalice cells, are interspersed among the ciliated and columnar cells throughout the epithelium. They appear to be formed by a modification of the columnar cell. They form granules which consist of mucin or mucigen inside the cell and in the upper part of the cell, while the nucleus is pressed down towards the base [46]. Like ciliated cells, the distribution of these cells has been mapped where the density of goblet cells is higher in the posterior part of the nose than the anterior [44]. The average number of goblet cells is about 4.000-7.000 cells per mm^2^. These cells are mucin-secreting cells or unicellular glands. Their contribution to the volume of nasal secretions is probably small, compared to submucosal glands, but little is known about their release mechanisms [16]. 

Goblet cells are not under the control of the sympathetic nervous system, like the glands, but respond to irritants, microenviroments or enterotoxins e.g. from Escherichia coli or Vibrio cholerae as well as other factors. The number of goblet cells increases during chronic irritation of the nasal passage. About two-third of the apical part of these cells, is filled with membrane-bound mucin secretory granules that have accumulated there. Once secreted the mucous forms a luminal lining lying on top of the glycocalyx of the microvilli. The mucous lubricates the mucosal surface and forms a barrier which protects the mucosal epithelium from potentially noxious intraluminal substances.

### Olfactory Cells

**Fig. (22):**
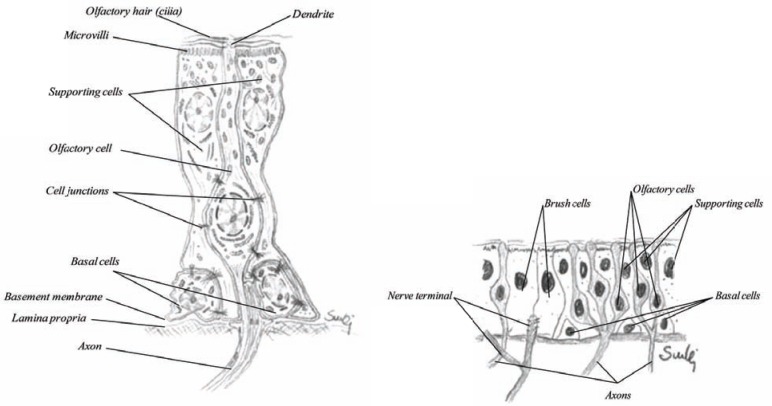
The structure of an olfactory cell together with supporting cells and basal cells (left) and olfactory mucosa (right).

The olfactory cells (Fig. **22**) are bipolar neurons located in the olfactory region of the nose and the vomeronasal organ. The olfactory epithelium is pseudostratified columnar epithelium with basement membrane like the respiratory epithelium. The epithelium is about 60 µm in height and has a slight yellow-brownish color due to the supporting cells also called the sustentaculum cells [48]. The olfactory cells are transducers of chemical sensations into neural signals. The mechanism of smell is still not fully understood, but it is known that the plasma membrane of the olfactory cells act as the actual site of chemoreception. Olfactory cells are so specialized that they need a set of supporting cells (Fig. **22**) to tend to their needs and protect them, these cells are actually sealed to the apex of the olfactory cells. The main body of the olfactory cell is therefore well isolated from the surroundings. To the apical surface stretched a dendrite, about 1 µm thick and the part expressed on the surface is called dendrite or olfactory knop, wherefrom about 6-10 cilia may be seen. The olfactory cilia are very long, over 50 µm having same microstructure as the cilia with microtubule. They are covered with mucus and float on the epithelial surface. These cilia are not motile. From the base, the ciliated cell is exposed through the axon (about 0,2 µm thick). Numerous axons are then bundled together to form so-called filia olfactoria, which may be seen going in the cribriform plate into the olfactory lobe of the brain. As shown in (Fig. **4**) there are numerous axons from other olfactory cells that go through each foramina and give rise to the olfactory nerve. Various studies have shown that absorption may also occur through the olfactory region. In many animals, however, such as the rabbit and the dog, this region will not be easily exposed by the drug. 

### Supporting Cells

The olfactory cells require supporting cells called sustentaculum cells (Fig. **22**). They are the most numerous cells in the olfactory epithelium and they provide metabolic and physical support to the olfactory cells as well as protection. They are known to possess lipofuscin granules. As mentioned above, the olfactory cells are sealed to these supporting cells, but not with gap and tight junctions. They are similar to the columnar cells, with numerous microvilli, but abundant of mitochondria. The nuclei of these cells are usually located more apically than other epithelial cells.

### Brush Cells

The olfactory epithelium as well as the vomeronasal organ present a small number of brush cells. They have large microvilli at their apical surface, but the basal surface is in synaptic contact with nerve fibers that penetrate the basal membrane. These nerve fibers are terminal branches of the trigeminal nerve (fifth cranial nerve), that has a more sensation function than olfaction [49]. Their function is receptor cells of general sensation such as irritation or other form of sensory stimulation of the mucosa.

## CELL JUNCTIONS

The epithelial membrane is tightly joined to form a close functional unit. The cells adhere to one another by specialized points of contacts between their adjacent plasma membranes called cell junctions. These junctions are able to prevent the movement of molecules between some cells but are also able to provide channels for molecules to communicate between others. 

Apically in the epithelial cells are junctional complexes called zona occludens also called tight junctions, which completely encircle the cells. A transmembrane protein, occludin, is the sealing protein, where the cytoplasmic portion of occludin is associated with zona occludens proteins ZO-1, ZO-2 and ZO3. These proteins are regulated by various cytokines and chemokines. Some pathogens like cytomegalovirus and cholera toxin interfere with ZO-1 and ZO-2, causing the junction to become permeable. This junction prevents the passage of most molecules between cells, because the adjacent membranes are almost fused together. It also restrict the passage of the cell own substances to be transported from the lateral site to the apical site and vice versa. Using an active transport, this junction has been found to play an essential role in the selective passage of substances from one side of an epithelium to the other. 

Two types of junctions are found, bearing plaque, a layer of dense proteins in the cytoplasm. One is the adherens junctions and the other is the desmosomes (macula adherens). The adherens junctions are connected to the cytoskeleton by actin-containing microfilaments, where the membranes are separated by an intercellular space of about 20 nm [26]. The transmembrane glycoproteins, E-cadherin, in the plaque of one cell cross this intracellular space through catenin and connect with the transmembrane proteins of an adjacent cell and establish the adhesion between the two cells via a cadherin-catenin complex, a Ca^2+^ ion dependent adhesion. These junctions form extensive zones called adhesion belt that completely encircle the cell, strengthening the ability to resist separation. Although the zonula occludens involves a fusion of adjoining cell membranes, their resistance to mechanical stress is limited.

The other plaque-bearing cell junctions are called macula adherens or desmosomes. They are scattered over the adjacent membrane and form strong attachments between the cells [50]. The membranes of adjacent cells at a desmosome are separated by an intercellular space of about 22-24 nm [26]. As with adherens junctions, the cells are anchored to each other by transmembrane glycoproteins, mainly desmogleins and desmocollins, also Ca^2+^-dependent adhesion molecules. There adhesions form a continuous cadherin zipper in the area of the desmosome. The filaments of desmosomes are, however, extended across the cytoplasm of the entire cell. This arrangement contributes to the overall stability of the tissue by connecting the cytoskeleton of adjoining cells. The desmosomes may also be connected to an extracellular material such as the basement membrane. In that case they are called hemidesmosomes. Hemi-desmosomes occur in epithelia that require strong, stable adhesion to the connective tissue. Columnar cells do not have these hemidesmosomes [16].

Gap junction is another type of junction between adjacent cells belonging to so-called communicating junctions, leaving an intercellular space of about 2 nm between them. The gap consists of an accumulation of transmembrane channels or pores in a tightly packed array. The proteins forming the tunnels in the membrane are called connexons. The tunnels are fluid filled where ions and small molecules such as glucose and amino acids, can pass directly from the cytoplasm of one cell into the cytoplasm of the adjacent cell, bringing messages back and forth. Electrical conductance studies show that if no gap junctions are between cells, the current flow is low due to high electrical resistance to the membrane, where the opposite is the situation when gap junctions are found between cells. 

Epithelial cells are anchored to the basement membrane to maintain the integrity of the epithelium. Two major anchoring junctions are found. First the focal adhesion, which anchors the actin filaments of the cytoskeleton to the basement membrane and the hemidesmosomes, described here above. The focal adhesions play the major role in the epithelia such as migration of epithelial cells in would repair. Their main transmembrane proteins are integrins, which are concentrated in clusters in the areas where the junctions are found.

## Figures and Tables

**Table 1.** The Architecture of the Nose (Anatomical Facts)

Factor	Value
Length	14 cm
Height	5 cm
Volume	15 mL
Volume per nostril (administered)	150 µL
Surface area	160 cm^2^
-including microvilli	9.6 m^2^
Olfactory region	8 cm^2^
-including microvilli	0.3 m^2^

**Table 2.** The Architecture of the Nose (Anatomy)

Factor	Branches
Neuronal supply	6
Arterial supply	6
Venous draining	5
Lymph draining	4

**Table 3.** The Anatomy of the Nasal Cavity

Factor	Number
Cell types	7
Respiratory region	4
Olfactory region	6
Glands types	4
Respiratory region	2
Olfactory region	2
Total number of glands (adult)	90.000
Openings (drains) to nasal cavity	7
from sinuses	5
